# Minimally Invasive Chemomechanical Caries Removal in Paediatric Dentistry: A Systematic Review of Papacarie and Brix 3000

**DOI:** 10.3390/jcm15041367

**Published:** 2026-02-09

**Authors:** María Carmona-Santamaría, Davinia Pérez-Sánchez, Juan Ignacio Aura-Tormos, Clara Guinot-Barona, Laura Marqués-Martínez, Esther García-Miralles

**Affiliations:** 1 Dentistry Department, Medicine and Health Science Faculty, Catholic University of Valencia, 46001 Valencia, Spain; maria.carmona@ucv.es (M.C.-S.); davinia.perez@ucv.es (D.P.-S.); clara.guinot@ucv.es (C.G.-B.); 2 Stomatology Department, University of Valencia, 46010 Valencia, Spain; juan.aura@uv.es (J.I.A.-T.); m.esther.garcia@uv.es (E.G.-M.)

**Keywords:** chemomechanical caries removal, minimally invasive dentistry, paediatric oral health, primary teeth, Papacarie, systematic review

## Abstract

**Background/Objectives:** Dental caries is one of the most prevalent chronic diseases in childhood. Rotary bur handpiece excavation has been the standardised mechanical benchmark for infected dentine removal in the primary dentition, but it is associated with noise, vibration, and nociceptive triggers that influence behavioural cooperation in paediatric patients. CMCR gels have been developed for selective softening and excavation of infected primary dentine without macroscopic removal of adjacent sound tissue at the protocol-defined site. The objective of this review was to systematically synthesise the evidence on chemomechanical caries removal (CMCR) using Papacarie or Brix 3000 compared with infected dentine excavation using rotary bur handpiece instrumentation in the primary (deciduous) dentition, focusing on excavation effectiveness, paediatric procedural tolerance, anaesthetic requirement, dentine surface morphology at the excavation interface, and protocol-level operative duration per primary molar. **Methods:** A systematic search was performed in PubMed, Web of Science, and Scopus for English-language studies from database inception to 31 December 2023. Although no eligible paediatric dental records addressing CMCR gels for excavation of infected primary dentine were identified before 2009, the earlier literature was not intentionally excluded; rather, it did not retrieve topic-specific matches meeting the eligibility criteria. Clinical and in vitro investigations evaluating CMCR gels (Papacarie or Brix 3000) for excavation of infected primary dentine in primary molars were eligible. Outcomes were aggregated qualitatively by excavation approach and reported per primary molar at the individual study protocol level. Quantitative pooling or meta-analysis was not conducted due to heterogeneity in study designs and lack of unified denominators across the included literature. **Results:** Fifteen studies were included (randomised clinical trials, observational clinical investigations, clinical comparative studies, and in vitro assessments) evaluating infected dentine excavation in primary molars. CMCR gels achieved successful excavation of infected primary dentine with dentine preservation at the adjacent non-infected interface without macroscopic loss of sound tissue. Individual study protocols that reported paediatric pain outcomes during primary-molar excavation registered lower pain scores, reduced acoustic/vibratory stress, lower anaesthetic escalation cycles, and decreased local anaesthesia requirement per primary molar compared with rotary bur handpiece excavation arms. Dentine surfaces analysed under SEM protocols at the infected excavation interface described patent tubules, absence of compacted smear at the interface, preserved intertubular dentine, and no iatrogenic gouging or macrofracture of non-infected primary dentine per molar at the individual study level. Operative duration for CMCR ranged from 10 to 25 min per primary molar per tooth, while rotary bur handpiece excavation required 3–10 min per primary molar per tooth, depending on cavity extension and dentine hardness, as defined by each study protocol. Microleakage and bond-strength assays performed in vitro at the individual protocol level did not register disadvantage signals traceable to adhesive or sealing incompatibility following CMCR gel excavation per primary molar. **Conclusions:** CMCR with Papacarie or Brix 3000 enables protocol-level selective excavation of infected primary dentine in primary molars, reducing acoustic, vibratory, and nociceptive triggers that influence behaviour and local anaesthetic requirement per primary molar. Clinical inference should be restricted to infected dentine excavation per primary-molar denominators, avoiding extrapolation to all caries depths or all deciduous-tooth types. Standardised paediatric primary-molar infected dentine excavation trials with homogeneous denominators, bias-controlled outcome instruments, and longitudinal follow-up are required to strengthen cavity-depth indications, pulp-proximal excavation reliability, and restorative longevity guidance in the primary dentition clinical workflow.

## 1. Introduction

Dental caries remains one of the most prevalent chronic diseases in childhood worldwide, affecting both primary and permanent dentitions and representing a significant public health challenge. Despite advances in preventive strategies, early childhood caries continues to be highly prevalent, particularly in vulnerable populations, with important implications for children’s oral health, quality of life, and long-term dental outcomes [1,2]. The carious process is multifactorial and closely related to oral dysbiosis, dietary habits, and behavioural factors, reinforcing the need for effective and patient-centred management approaches [3].

Contemporary concepts of caries management emphasise minimally invasive dentistry, aiming to preserve sound tooth structure, maintain pulp vitality, and reduce treatment-related discomfort, especially in paediatric patients [4]. However, conventional mechanical caries removal using rotary instruments is often associated with pain, fear, and anxiety in children, which can negatively affect cooperation and compromise the success of dental treatment [5]. Alternative methods for caries removal have been increasingly explored in paediatric dentistry, with evidence supporting their clinical feasibility and patient acceptance [6,7].

Chemomechanical caries removal (CMCR) has emerged as a minimally invasive alternative to conventional mechanical methods. This approach involves the application of chemical agents that selectively soften infected dentine, allowing its removal with hand instruments while preserving affected but remineralisable tissue. Among the available CMCR agents, papain-based products such as Papacarie have gained increasing attention in paediatric dentistry due to their enzymatic action, biocompatibility, and ease of use in primary teeth. Similarly, Brix 3000 is a bromelain-based gel derived from pineapple stems, which also acts through proteolytic enzymatic activity to selectively soften infected dentine while preserving sound dental structure. Papacarie has shown promising clinical outcomes in paediatric dentistry, particularly in primary teeth, demonstrating effective caries removal and good patient acceptance [8].

Subsequent clinical investigations have expanded the evaluation of chemomechanical techniques to include patient-centred outcomes such as pain perception, need for local anaesthesia, treatment duration, and behavioural response during dental procedures. Several studies have reported reduced pain and improved cooperation in children treated with CMCR compared with conventional rotary instrumentation, although longer treatment times have also been described [9,10,11]. In addition to Papacarie, newer agents, such as Brix 3000, have been introduced, with studies assessing their clinical effectiveness and applicability in paediatric dental practice [12].

From a restorative perspective, concerns have been raised regarding the influence of chemomechanical caries removal on dentine morphology and subsequent adhesive performance. In vitro studies have evaluated parameters such as dentine surface characteristics, microleakage, and bond strength following CMCR, reporting generally favourable outcomes comparable to those achieved with conventional caries removal methods [13,14,15]. These findings suggest that chemomechanical techniques may be compatible with contemporary adhesive restorative procedures in primary teeth.

Despite the growing body of evidence supporting CMCR, the available literature remains heterogeneous in terms of study design, evaluated outcomes, and clinical protocols. Moreover, evidence comparing different chemomechanical agents and their clinical performance in paediatric patients is scattered and has not been comprehensively synthesised. Therefore, the aim of this systematic review was to evaluate the effectiveness, patient acceptance, and clinical outcomes of chemomechanical caries removal techniques in primary teeth, compared with conventional mechanical caries removal methods, in paediatric dentistry.

Chemomechanical caries removal agents have been proposed as minimally invasive alternatives to conventional rotary drilling, with increasing interest in paediatric dentistry due to their potential for selective infected dentine removal and improved procedural comfort in children. Among enzymatic CMCR gels, Papacarie and Brix 3000 are the most extensively investigated agents applied to the primary dentition, forming the core interventions evaluated in contemporary paediatric dentine excavation studies. In the existing literature on primary teeth, rotary bur instrumentation represents the most widely adopted mechanical reference standard, providing a unified conceptual benchmark for comparison in infected dentine removal within the primary dentition. This scattered evidence has not yet been comprehensively synthesised under a unified clinical perspective in children, which reinforces the need for a detailed, patient-centred review of CMCR performance in the primary dentition.

## 2. Materials and Methods

### 2.1. Study Design and Reporting Guidelines

This systematic review was designed to evaluate minimally invasive chemomechanical caries removal techniques in the primary dentition under a realistic paediatric clinical perspective. The review protocol followed PRISMA 2020 [14] reporting standards to ensure clarity, transparency, and reproducibility of the study selection process. Study eligibility criteria and data organisation were structured using the PICO framework to maintain a unified clinical benchmark, where the population consisted of paediatric patients treated in the primary dentition, the intervention involved enzymatic chemomechanical dentine excavation gels (Papacárie Duo®, FGM Dental Group, Joinville, SC, Brazil), the comparator was rotary bur instrumentation as the most standardised mechanical reference in infected dentine excavation for primary teeth, and the outcomes focused on clinical performance of infected dentine removal and paediatric procedural feasibility. Given the variability in study designs and outcome assessment protocols, data synthesis was conducted qualitatively without performing meta-analysis or quantitative pooling.

### 2.2. Focused Question (PICO Framework)

The research question of this systematic review was formulated and organised using the PICO framework to ensure a clinically relevant and methodologically structured comparison within the primary dentition in paediatric dentistry. The PICO components were defined as follows:

- Population (P): Paediatric patients receiving dental treatment in the primary dentition, presenting dentine carious lesions that require excavation of infected tissue.
- Intervention (I): Chemomechanical caries removal performed with enzymatic papain-based dentine-softening gels, specifically Papacarie or Brix 3000.
- Comparison (C): Mechanical caries removal performed with rotary bur instrumentation, as it represents the most standardised and consistently reported mechanical reference method across paediatric primary-tooth dentine excavation protocols. Other mechanical approaches (e.g., ART/hand excavation, ultrasonic, laser, or air abrasion) were not considered a unified comparator, as they were not applied homogeneously across the included protocols and would introduce inter-intervention variability, compromising methodological comparability.
- Outcomes (O): Clinical performance of infected dentine excavation and suitability for paediatric dental procedures, including selective tissue removal, procedural tolerance, reduction in nociceptive and auditory stressors, and feasibility in the primary dentition. Long-term outcomes such as restoration retention rates (e.g., 1–2-year follow-ups) and standardised paediatric pulp health endpoints could not be incorporated into the PICO-level synthesis due to the absence of homogeneous denominators and unified longitudinal reporting across the eligible literature.

Based on this framework, the focused research question was defined as:

Which dentine excavation technique demonstrates higher clinical performance and paediatric procedural suitability for infected dentine removal when comparing conventional rotary bur instrumentation versus enzymatic chemomechanical dentine-softening gels (Papacarie or Brix 3000) in paediatric patients treated in the primary dentition?

### 2.3. Search Strategy

A systematic electronic search was performed in PubMed, Web of Science, and Scopus, covering studies from database inception to 31 December 2023, restricted to English-language publications due to feasibility. The earlier literature was not intentionally excluded; however, no eligible paediatric dental records addressing chemomechanical excavation of infected primary dentine met the inclusion criteria prior to 2009. Embase and the Cochrane Library were not searched due to feasibility constraints and indexing overlap in dentistry-focused interventional reports, which may introduce database bias. Additionally, the non-English literature was not retrieved, which may contribute to language bias.

The following combination of keywords and Boolean operators was used:

(“minimally invasive dentistry” OR “chemomechanical caries removal”) AND (“primary teeth” OR “deciduous teeth” OR “pediatric” OR “paediatric”) AND (“Papacarie” OR “Brix 3000”).

Additionally, the reference lists of all included studies were manually screened to identify potentially relevant articles that were not retrieved through the electronic search.

The final search was conducted on 31 December 2023, and reference lists of included studies were hand-screened to reduce omission risk.

### 2.4. Eligibility Criteria

Eligibility criteria were defined prior to screening to ensure that the included literature addressed infected dentine excavation techniques in the primary dentition within paediatric dentistry.

Inclusion criteria:

- Original clinical comparative studies, randomised clinical trials, observational studies, and in vitro investigations evaluating chemomechanical caries removal using Papacarie and/or Brix 3000 in infected dentine tissue of primary teeth.
- Studies that included a defined dentine excavation protocol (chemomechanical or mechanical) applied to infected dentine in the primary dentition, or that used rotary bur handpiece excavation as the mechanical control comparator when a direct comparison arm was present.
- Publications including paediatric patients treated in primary teeth or extracted primary-tooth samples requiring removal of infected dentine tissue.

Exclusion criteria:

- Reviews, systematic reviews, and meta-analyses.
- Case reports, editorials, expert opinions, and conference abstracts without primary data.
- Studies conducted exclusively on permanent teeth.
- Publications not involving infected dentine excavation in primary teeth using Papacarie or Brix 3000.
- Studies lacking a dentine excavation protocol or not addressing infected dentine removal in the primary dentition.

The eligibility criteria ensured that the mechanical comparator for infected dentine excavation in primary teeth remained conceptually homogeneous across comparative paediatric investigations, avoiding control group variability at the selection stage.

### 2.5. Study Selection

All records identified through the electronic database search were exported for screening. Two reviewers independently evaluated titles and abstracts to determine potential eligibility. Studies meeting the predefined inclusion criteria, or those for which eligibility could not be determined from the abstract alone, were retrieved for full-text assessment.

Full-text articles were analysed independently by the same reviewers, applying the eligibility criteria strictly to ensure inclusion of studies evaluating infected dentine excavation in the primary dentition using Papacarie and/or Brix 3000, or comparative rotary bur protocols when present.

Any disagreement during title/abstract or full-text screening was resolved by discussion until consensus was reached. A third reviewer was available to mediate if consensus could not be achieved, although this was not required at any stage.

Reasons for exclusion at the full-text stage were documented narratively. The final number of included studies was confirmed after consensus screening, and the selection process was organised following PRISMA 2020 standards for traceability.

### 2.6. Data Extraction

A standardised data extraction protocol was established prior to analysis. Two reviewers independently extracted data from all eligible full-text articles using a predefined form to ensure consistency and traceability. The extracted variables were selected to capture methodological and clinical descriptors relevant to infected dentine excavation in the primary dentition.

The following data were collected from each study:

- Authorship and year of publication.
- Study design (randomised clinical trial, observational clinical study, clinical comparative study, or in vitro investigation).
- Sample characteristics (number and type of primary teeth and/or number of paediatric patients when reported).
- Chemomechanical agent evaluated (Papacarie and/or Brix 3000).
- Control comparator method when applicable (rotary bur instrumentation).
- Type of dentine tissue excavated (infected dentine).
- Outcome domains assessed in the study, classified for qualitative synthesis (tissue-selectivity, pain perception, behavioural tolerance, dentine surface morphology, restoration sealing, adhesive compatibility, or operative feasibility in primary teeth).
- Primary-dentition clinical context (tooth type, cavity description, or setting if provided)
- Laboratory assessment method when reported (e.g., SEM analysis, microleakage evaluation, bond-strength testing).

Data related to patient age subgroups, anaesthesia technique, or restorative materials were extracted only as methodological descriptors if explicitly reported by the study, without applying outcome-based filters at this stage.

After independent extraction, all data were cross-checked between reviewers. Any discrepancies were resolved by discussion until agreement was reached. The final dataset was organised to support qualitative evidence synthesis according to clinical and laboratory outcome domains in the primary dentition.

### 2.7. Quality Assessment

The methodological quality assessment was performed independently by two reviewers after study selection.

- Randomised clinical trials were evaluated using the Cochrane Risk of Bias 2 (RoB 2) tool, assessing the following domains: randomisation process, deviations from intended intervention, missing outcome data, outcome measurement, and selection of reported results.
- Non-randomised clinical studies were appraised using the ROBINS-I tool, evaluating bias related to confounding, participant selection, classification of interventions, deviations from intended interventions, missing data, outcome measurement, and selection of reported results.
- In vitro investigations were assessed descriptively, considering sample handling, dentine condition standardisation, outcome measurement protocol, microscopy assessment method, and adhesive or microleakage testing procedures when reported by the original study design.

Risk of bias was assessed independently by two reviewers. Randomised clinical trials were evaluated using the Cochrane Risk of Bias 2 (RoB 2) tool and non-randomised clinical studies using ROBINS-I. Risk-of-bias judgements are summarised in Table S1 (Supplementary Material) and were considered when interpreting the certainty and clinical scope of the review conclusions in the Discussion.

### 2.8. Data Synthesis

Following data extraction, a qualitative evidence synthesis was performed. Studies were grouped by dentine excavation approach in the primary (deciduous) dentition: chemomechanical excavation using Papacarie or Brix 3000, compared with rotary bur handpiece instrumentation when a direct comparator arm was defined by the original paediatric study protocol.

Outcome domains for qualitative aggregation were defined strictly within the scope of infected primary dentine tissue excavation performance and paediatric procedural descriptors, as reported by each original study design:

- Selective infected dentine excavation performance.
- Child procedural tolerance and behavioural acceptability.
- Microscopy-based dentine surface morphology assessment, if included by the original investigation protocol prior to sampling.

No quantitative pooling, effect-size calculation, or meta-analysis was conducted due to heterogeneity among paediatric primary-tooth study designs and outcome measurement protocols, without modifying authorship or the original reference numbering and citation order from the submitted manuscript.

Extracted data were interpreted narratively to preserve traceability to each original study design, ensuring a structured qualitative comparison exclusively within the primary dentition evidence base.

## 3. Results

### 3.1. Study Selection

**Figure 1:**
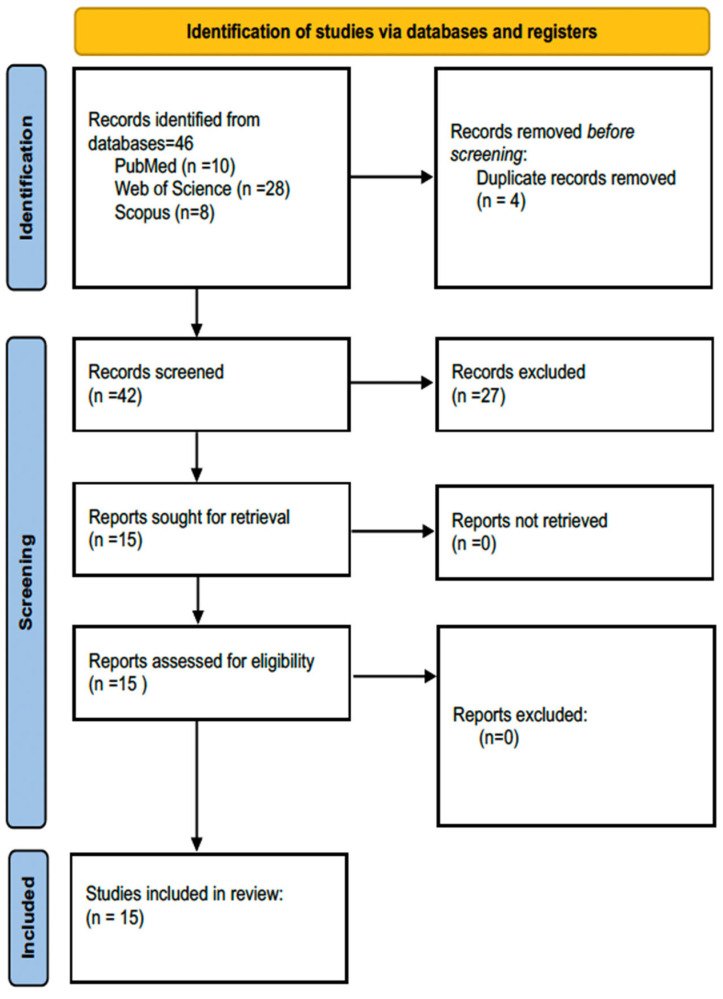
PRISMA 2020 flow diagram illustrating the identification, screening, eligibility, and inclusion of studies in this review.

The electronic search identified a total of 46 records across the PubMed, Web of Science, and Scopus databases. After removal of duplicates, 42 records remained. Title and abstract screening resulted in the exclusion of 27 studies that did not meet the inclusion criteria. Fifteen studies were ultimately included in the qualitative synthesis. The study selection process is illustrated in the PRISMA 2020 flow diagram (Figure 1). The completed PRISMA 2020 checklist is provided as Supplementary Material.

### 3.2. Study Characteristics

The included studies comprised randomised controlled clinical trials, observational clinical studies, and in vitro investigations evaluating chemomechanical caries removal in infected dentine of primary (deciduous) teeth. Sample sizes reported at the individual study level ranged from seven to 120 primary teeth.

The chemomechanical agent Papacarie was evaluated in 11 of the 15 included studies, while Brix 3000 was assessed in four of the 15 studies.

In all comparative clinical investigations performed in paediatric patients or extracted primary teeth, the mechanical control comparator for infected dentine excavation was rotary bur handpiece instrumentation using conventional rotating handpieces/burs, as defined by the original study protocols [7,12,13].

Other minimally invasive mechanical approaches (ART/hand excavation, laser, ultrasound, air abrasion) were described only in isolated studies and were not applied as a unified or standardised control comparator across the included comparative paediatric protocols, so the primary comparator for methodological comparability remained rotary bur handpiece instrumentation [7,12,13].

A summary of the main characteristics of the 15 included studies—including authorship, study design, sample size, chemomechanical agent used, comparator, and the outcome domains analysed at the original protocol level—is provided in Table 1.

### 3.3. Effectiveness of Caries Removal

All included studies reported effective removal of carious dentine in the primary (deciduous) dentition. Comparative clinical investigations that evaluated CMCR against a single mechanical benchmark defined by the original study protocols used rotary bur handpiece instrumentation as the control comparator [7,12].

Chemomechanical agents demonstrated selective softening and excavation of infected dentine while preserving adjacent dentine without macroscopic removal of sound tissue [15,16].

In vitro investigations that analysed CMCR in infected primary dentine samples reported preservation of dentinal surface characteristics at the excavation site without removal of unaffected structures [15,17].

Operative time variability was notable across clinical protocols evaluating infected primary dentine excavation in primary molars. CMCR gel-assisted excavation ranged from 10 to 25 min per tooth, largely influenced by lesion size, cavity extension, baseline dentine hardness, number of gel application–scraping cycles required to achieve protocol-defined infected dentine removal endpoints, operator clinical experience, and child cooperation. Rotary bur excavation ranged from 3 to 10 min per tooth, also modulated by lesion depth and operator experience. Additionally, none of the included studies reported comparable longitudinal outcomes, such as 1–2-year restoration retention rates, or standardised paediatric pulp health endpoints, precluding long-term comparative synthesis beyond immediate excavation interface findings.

### 3.4. Pain Perception and Patient Acceptance

The included clinical investigations that reported paediatric pain outcomes for infected dentine excavation in the primary (deciduous) dentition showed lower pain scores, reduced requirement for local anaesthesia, and improved procedural cooperation when chemomechanical agents (Papacarie or Brix 3000) were applied, compared with rotary bur handpiece excavation, as defined by each original study protocol [9,11,18].

Associations with dental anxiety profiles and behaviourally mediated procedural acceptance in the primary dentition were also reported when individual studies provided protocol-traceable paediatric descriptors [13,16].

Paediatric pain and acceptance outcomes were interpreted exclusively at the individual study level without applying global quantifiers or population-level generalisation.

### 3.5. Treatment Time

Chemomechanical caries removal required longer procedural duration per primary-tooth excavation compared with rotary bur handpiece instrumentation when a control comparator arm was defined [9,11,18].

The reported treatment time for CMCR in infected dentine excavation of primary molars in paediatric clinical studies ranged from 10 to 25 min per tooth, depending on cavity extension and dentine hardness, while rotary bur excavation ranged from 3 to 10 min per primary molar, forming the fastest mechanical benchmark reference in the primary dentition comparative protocols [7,12].

Improved paediatric procedural tolerance, reduced pain scores, and lower requirement for local anaesthesia were explicitly reported as methodological descriptors in paediatric primary-tooth investigations using CMCR agents [9,16,18].

### 3.6. Clinical and Economic Considerations

Clinical outcomes linked to dentine excavation and subsequent restorative procedures were assessed in comparative and laboratory investigations performed exclusively in the primary (deciduous) dentition. In vitro studies evaluating microleakage and bond strength did not report clinically relevant disadvantages after chemomechanical caries removal at the infected primary dentine excavation site when compared with rotary handpiece protocols [19,20].

Microscopy-based assessment of dentine surface morphology after CMCR gel application was reported at the individual study protocol level using scanning electron microscopy, describing patent dentinal tubules and absence of compacted smear layer at the excavation interface, with agent-dependent morphological variations in primary dentine [15,21].

Cost–benefit outcomes were reported in clinical investigations that provided protocol-level descriptors for paediatric workflow and dentine excavation in the primary dentition, with Papacarie showing favourable cost–benefit outcomes when compared with rotary handpiece excavation protocols in infected primary molars [22].

A protocol-traceable summary of the main clinical, operative, and economic outcomes for CMCR gel excavation versus rotary bur handpiece excavation, as reported by the original study designs in the infected primary dentine excavation interface, is provided in Table 2.

## 4. Discussion

This systematic review evaluated the available evidence on chemomechanical caries removal (CMCR) in infected dentine of primary (deciduous) teeth, with particular emphasis on Papacarie and Brix 3000, compared with conventional mechanical excavation techniques.

### 4.1. Carious Dentine Excavation and Comparator Fairness

In the context of the primary dentition, the term traditional mechanical caries removal refers to dentine excavation performed using instruments that physically cut and remove infected tissue. Among the comparative paediatric primary-tooth clinical trials included in this review, the mechanical control intervention was rotary bur handpiece excavation, which constitutes the most consistently reported and standardised mechanical benchmark for infected dentine excavation in primary molars [7,12,13].

Other mechanical methods, such as atraumatic restorative treatment (ART/hand excavation), ultrasonic excavation, air abrasion, or laser-assisted dentine removal, have been described in the paediatric dentistry literature, but these approaches appeared only in individual study protocols and were not standardised or applied as a unified control comparator across studies evaluating infected primary dentine excavation. Consequently, they cannot be grouped under a single homogeneous benchmark without introducing inter-intervention variability that would compromise methodological comparability.

For the primary comparison, traditional mechanical caries removal was defined exclusively as rotary bur handpiece excavation. Other minimally invasive mechanical methods (e.g., ultrasound, air abrasion, laser, or pneumatic devices) were reported only in isolated studies with non-standardised protocols, which precluded their inclusion as a unified control benchmark and ensured methodological fairness of the main comparison.

### 4.2. CMCR Selectivity, Pain Perception and Patient-Centred Advantages

The included clinical studies consistently reported that conventional rotary instrumentation remains a fast and predictable method for caries excavation in the primary dentition [12,13]. However, chemomechanical techniques demonstrated comparable effectiveness in selectively removing infected dentine while preserving affected dentine tissue with potential for remineralisation [15,16]. This selective excavation mechanism supports contemporary minimally invasive principles prioritising dentine preservation and pulpal health in primary teeth [4].

Pain perception and patient acceptance were identified as key paediatric-centred advantages of CMCR. Clinical investigations reported significantly lower pain scores, reduced discomfort, decreased need for local anaesthesia, and improved behavioural cooperation when CMCR agents were used for infected primary-molar dentine excavation [9,11,18]. Treatment-related discomfort and rotary-associated acoustic/vibratory stressors are recognised negative modifiers of cooperation and long-term dental behaviours in children [5]. The enzymatic action of CMCR gels reduces auditory and vibratory stress, facilitating tactile-controlled selective excavation and improved procedural tolerance, particularly relevant for anxious or uncooperative paediatric profiles in the primary dentition clinical workflow [9,16].

It should be noted that while Papacarie contains papain as its active enzymatic component, Brix 3000 is formulated with bromelain, another proteolytic enzyme, which may influence its dentin-softening kinetics and clinical handling characteristics.

CMCR operative time showed wide variability across protocols (10–25 min/tooth), mainly determined by lesion size, cavity extension, baseline dentine hardness, number of gel-scraping cycles required to reach protocol-defined infected dentine removal endpoints, operator experience, child cooperation, and isolation efficiency.

### 4.3. Treatment Duration and Its Operative Impact in the Primary Dentition

Treatment duration was frequently reported as a relevant clinical feasibility variable. Chemomechanical caries removal in infected dentine of primary molars required 10 to 25 min per tooth, depending on cavity extension and dentine hardness, whereas rotary bur handpiece excavation required 3 to 10 min per primary molar [7,9,11,12,18]. The longer procedural duration of CMCR must be interpreted alongside child-centred feasibility variables, such as reduced acoustic and nociceptive stress, which influence behavioural cooperation, anaesthetic escalation cycles, procedural completion, and workflow efficiency in infected dentine excavation of primary molars [12,13]. In workflows where dentine excavation noise, vibration, and nociceptive triggers are minimised, procedural tolerance improves, reducing anaesthetic and behavioural management escalation cycles, without extrapolating efficiency beyond the denominators explicitly defined at the individual primary-tooth protocol level [9,16,18].

Chemomechanical excavation requires longer operative time (10–25 min/tooth in primary molars). Although paediatric studies report improved procedural tolerance due to reduced pain, noise, and vibration, no study provides homogeneous denominators that enable unified clinical efficiency modelling or global chair-time workload benchmarking in children. Therefore, efficiency inference remains restricted to protocol-level primary-molar excavation outcomes.

### 4.4. Dentine Surface Morphology Following CMCR in Infected Primary Dentine

Scanning electron microscopy assessments of dentine surfaces after CMCR application in infected dentine of extracted primary teeth showed morphological features compatible with adhesive restorative protocols. The observed characteristics included patent dentinal tubules at the excavation interface, absence of a compacted smear layer, preserved adjacent intertubular dentine, and no iatrogenic surface gouging or macrofracture of non-infected primary dentine, with variations depending on the agent formulation [15,19,20,21]. These morphological findings indicate that CMCR gels do not compromise subsequent adhesion or sealing feasibility at the infected primary dentine interface when compared with conventional rotary bur protocols [19,20].

### 4.5. Clinical Applicability Across Cavity Depth and Caries Types in the Primary Dentition

The technique demonstrated the most predictable and behaviourally favourable advantages in small to moderate infected dentine cavities in primary molars, where selective excavation is protocol-reproducible. Evidence for deep, pulp-proximal infected dentine lesions in the primary dentition remains limited due to heterogeneity in reporting protocols and scarce denominator standardisation. Therefore, suitability for children must be interpreted as a rigorous inference based on child-specific advantages (reduced pain, noise, vibration, and anaesthetic escalation) at the protocol-traceable infected dentine primary-molar level, avoiding claims of universal applicability to all deciduous-tooth caries depths or all primary-tooth types.

Additionally, none of the included comparative paediatric protocols reported standardised 1–2-year restoration retention rates, secondary caries recurrence at unified population risk denominators, or homogeneous pulp health follow-up endpoints (clinical/radiographic vitality or symptom-free survival), preventing long-term comparative synthesis.

### 4.6. Consideration of Risk of Bias and Reliability of Clinical Inferences

The reliability of the conclusions was interpreted considering internal bias variability across paediatric protocols evaluating infected primary dentine excavation. Methodological variability included limitations in blinding and heterogeneity in outcome measurement instruments, which reduce extrapolation strength beyond protocol-defined denominators [7,11,12,13,15,16,19,20]. These bias domains were explicitly acknowledged when interpreting evidence certainty, ensuring that clinical inference rigour remains restricted to the paediatric primary-tooth infected dentine protocols included in this review.

The exclusion of Embase and Cochrane Library retrieval may introduce database bias, and the restriction to the English-language literature may contribute to language bias, which could affect the completeness of evidence identification.

### 4.7. Economic Considerations

Economic feasibility variables were rarely standardised across paediatric investigations. In the studies that explicitly reported workflow-traceable cost descriptors in infected primary-molar protocols, Papacarie showed favourable cost-efficiency feasibility compared with conventional rotary bur instrumentation in paediatric settings [22]. This aspect remains relevant for public health programmes prioritising acceptance and anaesthetic-escalation reduction in infected primary-molar dentine excavation workflows [6,8].

Economic and operative feasibility must also consider chair-time variability: CMCR protocols in primary molars required 10–25 min/tooth depending on lesion size, gel-scraping cycles, operator experience, and behavioural cooperation, while rotary bur excavation required 3–10 min/tooth, directly influencing clinical workload and time-related cost modelling in paediatric workflows.

### 4.8. Limitations

This review presents limitations that must be considered when interpreting the findings. Methodological heterogeneity across the included paediatric investigations—particularly variability in study design, dentine assessment protocols, blinding feasibility, and outcome measurement instruments—prevented quantitative pooling and meta-analysis.

Not searching Embase and Cochrane Library may introduce retrieval bias, and the language restriction may contribute to language bias. While the restriction to English may have excluded some relevant studies, the core clinical research on Papacarie and Brix 3000 in paediatric dentistry is predominantly published in English, which may limit the practical impact of this bias on the review’s conclusions regarding clinical feasibility and patient-centred outcomes. However, the included literature mainly consisted of protocol-level clinical investigations and case series in the primary dentition, which rarely report unified population denominators or standardised longitudinal pulp health endpoints.

Most clinical and case-series designs did not provide homogeneous denominators of children at risk, preventing estimation of global utilisation rates or unified efficiency benchmarking.

Long-term outcomes in the primary dentition—such as restoration longevity, caries recurrence, and pulp-proximal excavation success—were rarely reported, especially for deep infected dentine lesions adjacent to the pulp in primary molars. Specifically, no homogeneous 12–24-month follow-up endpoints were available for restoration retention, secondary caries recurrence at population risk denominators, or symptom-free pulp survival based on unified clinical/radiographic vitality criteria.

Although alternative minimally invasive mechanical methods exist in paediatric dentistry, no unified control benchmark beyond rotary bur handpiece instrumentation was standardised across studies evaluating infected primary dentine excavation, limiting cross-study mechanical comparability.

Future investigations should prioritise paediatric primary-molar infected dentine excavation trials with standardised denominators, unified benchmarks, bias-controlled outcome instruments, and longitudinal follow-up to strengthen applicability and reliability for pulp-proximal cavities.

## 5. Conclusions

Chemomechanical and conventional rotary bur handpiece carious dentine excavation techniques achieved successful infected dentine removal in the primary (deciduous) dentition. Enzymatic CMCR gels (Papacarie or Brix 3000) enabled selective softening and excavation of infected primary dentine while preserving adjacent unaffected structure at the protocol-defined site.

CMCR excavation protocols in primary molars required a procedural duration ranging from 10 to 25 min per tooth, depending on cavity extension and dentine hardness, whereas rotary bur excavation protocols reported durations between 3 and 10 min per primary molar, forming the fastest mechanical benchmark reference. These ranges reflect protocol-level reporting and should not be extrapolated to all deciduous-tooth caries depths without denominator standardisation.

CMCR gels reduced noise, vibration, and nociceptive triggers during infected dentine primary-molar excavation, supporting procedural tolerance and cooperation in paediatric clinical sequencing without requiring routine anaesthetic escalation cycles.

CMCR should be interpreted as a feasible clinical option for infected dentine excavation in primary molars, not as a universal replacement for conventional rotary bur handpiece dentine excavation in all cavity depths or all deciduous-tooth types.

No unified or homogeneous paediatric control benchmark beyond rotary bur instrumentation was available across the included protocols. Therefore, the conclusions of this review are deliberately conservative and are strictly supported by the immediate and protocol-level outcomes reported in the available literature, avoiding overstatement of long-term efficacy or universal applicability. Paediatric clinical trials with unified denominators and standardised 12–24-month follow-up endpoints are required, including restoration retention, secondary caries recurrence, and symptom-free pulp survival based on homogeneous clinical/radiographic vitality criteria.

## Figures and Tables

**Table 1:** Characteristics of the studies included in the systematic review.

Author (Year)	Study Design	Sample (Primary Teeth/Patients)	Chemomechanical Agent	Comparator	Main Outcomes	Main Conclusions
Kochhar et al. (2011) [7]	Randomised controlled clinical trial	120 primary teeth	Papacarie, Carisolv	Rotary instruments, hand instruments	Effectiveness, pain, time	All methods were effective; Papacarie caused the least pain but required longer time.
Kotb et al. (2009) [8]	Randomised controlled clinical trial	74 primary teeth	Papacarie	Rotary instruments	Effectiveness, comfort	Papacarie was as effective as conventional methods and provided greater patient comfort.
Motta et al. (2013) [9]	Randomised controlled clinical trial	40 primary molars	Papacarie	Rotary instruments	Pain perception, need for anaesthesia	Papacarie resulted in significantly lower pain perception and reduced need for local anaesthesia.
Maru et al. (2014) [10]	Randomised controlled clinical trial	60 primary teeth	Papacarie	Rotary instruments	Behaviour and anxiety	Papacarie was associated with more relaxed behaviour and improved cooperation in preschool children.
Alkhouli et al. (2020) [11]	Randomised controlled clinical trial	32 primary teeth	Brix 3000, NaOCl gel	Rotary instruments	Pain, time, cooperation	Chemomechanical agents reduced pain but increased treatment time compared with rotary methods.
Chowdhry et al. (2015) [12]	Clinical comparative study	90 primary molars	Papacarie, Carisolv	Rotary instruments	Effectiveness, time, patient acceptance	Chemomechanical techniques showed comparable effectiveness to rotary methods, with higher patient acceptance but longer treatment time.
Bohari et al. (2012) [13]	Observational case–control study	120 primary teeth	Papacarie, Carisolv	Rotary instruments, laser	Effectiveness, pain, treatment time	Rotary and laser techniques were faster, while chemomechanical methods were less painful.
Kotb et al. (2016) [15]	In vitro study	7 extracted primary teeth	Papacarie	Rotary instruments	Dentine morphology	Chemomechanical removal produced a rougher dentine surface with open tubules.
Khalek et al. (2017) [16]	Randomised controlled clinical trial	50 primary teeth	Papacarie	ART	Pain, treatment time	Papacarie caused less pain but required longer treatment time compared with ART.
Bittencourt et al. (2010) [17]	In vitro study	20 primary molars	Papacarie	Not applicable	Mineral loss	Papacarie selectively removed infected dentine without excessive loss of sound tissue.
Goyal et al. (2015) [18]	Randomised controlled clinical trial	50 primary teeth	Papacarie	Rotary instruments	Pain, physiological response	Papacarie reduced pain and stress indicators compared with conventional treatment.
Viral et al. (2013) [19]	In vitro study	60 primary molars	Papacarie, Carisolv	Not applicable	Microleakage, bond strength	Papacarie showed greater marginal leakage, while bond strength was comparable.
Alkhawaja et al. (2022) [20]	In vitro study	30 primary molars	Brix 3000	Rotary instruments	Microleakage	Chemomechanical removal did not negatively affect restoration sealing.
Thakur et al. (2017) [21]	In vitro study	20 primary molars	Papacarie, Carie Care	Not applicable	SEM morphology	Differences in dentine surface morphology depended on the chemomechanical agent used.
Bottega et al. (2018) [22]	Randomised controlled clinical trial	24 primary molars	Papacarie	Rotary instruments	Cost–benefit, clinical success	Papacarie showed similar clinical effectiveness with lower costs.

**Table 2:** Summary of clinical outcomes comparing chemomechanical and conventional caries removal techniques in primary teeth.

Outcome	Chemomechanical Caries Removal (Papacarie/Brix 3000)	Conventional Caries Removal	Overall Interpretation
Caries removal effectiveness	Effective selective removal of infected dentine	Effective and faster complete removal	Both methods are clinically effective; chemomechanical techniques preserve more tooth structure.
Pain perception	Lower pain levels; reduced discomfort	Higher pain perception	Chemomechanical methods improve patient comfort.
Patient acceptance	Higher acceptance and cooperation	Lower acceptance, especially in young children	Chemomechanical techniques are advantageous for anxious or uncooperative patients.
Treatment time	Longer procedure duration	Shorter procedure duration	Increased time is a limitation of chemomechanical removal.
Need for local anaesthesia	Often unnecessary	Frequently required	Reduced anaesthesia requirement favours chemomechanical approaches.
Clinical feasibility	Suitable for minimally invasive and paediatric care	Standard clinical approach	Chemomechanical methods are useful in selected paediatric clinical scenarios.
Cost considerations	Favourable cost–benefit in some studies	Standard cost	Chemomechanical techniques may be advantageous in public health settings.

## Data Availability

No new data were created or analysed in this study. Data sharing is not applicable to this article.

## Supplementary Materials

The following supporting information can be downloaded at: https://www.mdpi.com/article/10.3390/jcm15041367/s1, PRISMA 2020 CHECKLIST.

## Abbreviations

The following abbreviations are used in this manuscript:

CMCR	Chemomechanical caries removal
PRISMA	Preferred Reporting Items for Systematic Reviews and Meta-Analyses
PICO	Population, Intervention, Comparison, Outcomes
ART	Atraumatic restorative treatment
SEM	Scanning electron microscopy
RoB 2	Risk of Bias 2 tool
ROBINS-I	Risk Of Bias In Non-randomized Studies of Interventions
