# Supplementary material for: Minimally Invasive Chemomechanical Caries Removal in Paediatric Dentistry: A Systematic Review of Papacarie and Brix 3000
Source: J Clin Med. 2026 Feb 9;15(4):1367. doi: 10.3390/jcm15041367 (PMC12942050; doi:10.3390/jcm15041367)
Supplement: Supplementary file 1 [file jcm-15-01367-s001.zip › jcm-4085810-supplementary.pdf]

PRISMA 2020 CHECKLIST

Minimally Invasive Chemomechanical Caries Removal in Pediatric Dentistry: A Systematic Review of Papacarie and Brix 3000

TITLE

Item 1. Identify the report as a systematic review.

Location: Title

ABSTRACT

Item 2. Provide a structured summary in accordance with PRISMA 2020 for Abstracts.

Location: Abstract

INTRODUCTION

Item 3. Describe the rationale for the review in the context of existing knowledge.

Location: Introduction

Item 4. Provide an explicit statement of the objectives or questions the review addresses.

Location: Introduction

METHODS

Item 5. Specify the inclusion and exclusion criteria for the review and how studies were grouped for synthesis.

Location: Section 2.4 (Eligibility Criteria)

Item 6. Specify all information sources (e.g., databases) used to identify studies and the date when each was last searched.

Location: Section 2.3 (Search Strategy)

Item 7. Present the full search strategies for all databases, including any filters or limits used.

Location: Section 2.3 (Search Strategy)

Item 8. Specify the methods used to decide whether a study met the inclusion criteria, including how many reviewers screened each record and how disagreements were resolved.

Location: Section 2.5 (Study Selection)

Item 9. Specify the methods used to collect data from reports, including how many reviewers collected data and how discrepancies were resolved.

Location: Section 2.6 (Data Extraction)

Item 10a. List and define all outcomes for which data were sought.

Location: Sections 2.2 and 2.6

Item 10b. List and define all other variables for which data were sought.

Location: Section 2.6

Item 11. Specify the methods used to assess risk of bias in the included studies.

Location: Section 2.7 (Quality Assessment)

Item 12. Specify effect measures used for each outcome.

Location: Not applicable (qualitative synthesis only)

Item 13a. Describe the processes used to decide which studies were eligible for each synthesis.

Location: Section 2.8 (Data Synthesis)

Item 13b. Describe any methods required to prepare data for presentation or synthesis.

Location: Section 2.8

Item 13c. Describe methods used to tabulate or visually display results.

Location: Section 3.2; Tables 1 and 2

Item 13d. Describe methods used to synthesize results.

Location: Section 2.8

Item 13e. Describe any methods used to explore possible causes of heterogeneity.

Location: Not applicable

Item 13f. Describe any sensitivity analyses conducted.

Location: Not applicable

Item 14. Describe any methods used to assess risk of bias due to missing results.

Location: Not applicable

Item 15. Describe any methods used to assess certainty or confidence in the body of evidence.

Location: Not applicable

RESULTS

Item 16a. Describe the results of the search and selection process, including the number of studies screened and included.

Location: Section 3.1; Figure 1 (PRISMA flow diagram)

Item 16b. Cite studies that were excluded after full-text assessment, with reasons.

Location: Section 3.1

Item 17. Cite each included study and present its characteristics.

Location: Section 3.2; Table 1

Item 18. Present assessments of risk of bias for each included study.

Location: Section 2.7

Item 19. Present results of individual studies.

Location: Sections 3.3–3.6

Item 20a. Summarize the results of each synthesis.

Location: Sections 3.3–3.6; Table 2

Item 20b. Present results of any statistical heterogeneity analyses.

Location: Not applicable

Item 20c. Present results of sensitivity analyses.

Location: Not applicable

Item 21. Present assessments of risk of bias due to missing results.

Location: Not applicable

Item 22. Present assessments of certainty of evidence.

Location: Not applicable

DISCUSSION

Item 23a. Provide a general interpretation of the results in the context of other evidence.

Location: Section 4

Item 23b. Discuss limitations of the evidence included in the review.

Location: Section 4.1

Item 23c. Discuss limitations of the review processes used.

Location: Section 4.1

Item 23d. Discuss implications of the results for practice, policy, and future research.

Location: Sections 4 and 5

OTHER INFORMATION

Item 24a. Provide registration information for the review.

Location: Not registered

Item 24b. Indicate where the review protocol can be accessed.

Location: Not applicable

Item 24c. Describe any amendments to the protocol.

Location: Not applicable

Item 25. Describe sources of financial or non-financial support.

Location: Funding / Acknowledgments

Item 26. Declare any competing interests.

Location: Conflicts of Interest

Item 27. Describe availability of data, code, and materials.

Location: Data Availability Statement

From: Page MJ, McKenzie JE, Bossuyt PM, Boutron I, Hoffmann TC, Mulrow CD, et al. The PRISMA 2020 statement: an updated guideline for reporting systematic reviews. BMJ 2021;372:n71. doi: 10.1136/bmj.n71. This work is licensed under CC BY 4.0. To view a copy of this license, visit https://creativecommons.org/licenses/by/4.0/
